# Supplementary material for: Direct van der Waals simulation (DVS) of phase-transforming fluids
Source: Sci Adv. 2023 Mar 17;9(11):eadg3007. doi: 10.1126/sciadv.adg3007 (PMC10022896; doi:10.1126/sciadv.adg3007)
Supplement: Supplementary file 1 — Transformation matrices [file sciadv.adg3007_sm.pdf]

Science Advances
AAAS

Supplementary Materials for

Direct van der Waals simulation (DVS) of phase-transforming fluids

Tianyi Hu *et al*.

Corresponding author: Hector Gomez, hectorgomez@purdue.edu

Sci. Adv. **9**, eadg3007 (2023)
DOI: 10.1126/sciadv.adg3007

This PDF file includes:

Transformation matrices

Transformation matrices

$$\boldsymbol{A}_0 = \begin{bmatrix} 1 & 0 & 0 & 0 \\ u_1 & \rho & 0 & 0 \\ u_2 & 0 & \rho & 0 \\ u_3 & 0 & 0 & \rho \end{bmatrix}, \tag{A.1}$$

$$\boldsymbol{A}_1^{\mathrm{adv/p}} = \begin{bmatrix} u_1 & \rho & 0 & 0 \\ u_1^2 & 2\rho u_1 & 0 & 0 \\ u_1 u_2 & \rho u_2 & \rho u_1 & 0 \\ u_1 u_3 & \rho u_3 & 0 & \rho u_1 \end{bmatrix}, \tag{A.2}$$

$$\boldsymbol{A}_2^{\mathrm{adv/p}} = \begin{bmatrix} u_2 & 0 & \rho & 0 \\ u_1 u_2 & \rho u_2 & \rho u_1 & 0 \\ u_2^2 & 0 & 2\rho u_2 & 0 \\ u_2 u_3 & 0 & \rho u_3 & \rho u_2 \end{bmatrix}, \tag{A.3}$$

$$\boldsymbol{A}_3^{\mathrm{adv/p}} = \begin{bmatrix} u_3 & 0 & 0 & \rho \\ u_1 u_3 & \rho u_3 & 0 & \rho u_1 \\ u_2 u_3 & 0 & \rho u_3 & \rho u_2 \\ u_3^2 & 0 & 0 & 2\rho u_3 \end{bmatrix}, \tag{A.4}$$

$$\boldsymbol{A}_i^{p} = p_{,\rho} \boldsymbol{e}_{i+1} \otimes \boldsymbol{e}_i, \text{ (no sum on } i) \tag{A.5}$$

$$\boldsymbol{A}_i^{c} = \frac{\lambda \eta \rho \Delta \rho_{,i}}{\rho_{,i}} \boldsymbol{e}_{i+1} \otimes \boldsymbol{e}_i, \text{ (no sum on } i) \tag{A.6}$$

$$\boldsymbol{K}_{11} = \begin{bmatrix} 0 & 0 & 0 & 0 \\ 0 & 2\overline{\mu} + \overline{\lambda} & 0 & 0 \\ 0 & 0 & \overline{\mu} & 0 \\ 0 & 0 & 0 & \overline{\mu} \end{bmatrix}, \tag{A.7}$$

$$\boldsymbol{K}_{12} = \begin{bmatrix} 0 & 0 & 0 & 0 \\ 0 & 0 & \overline{\lambda} & 0 \\ 0 & \overline{\mu} & 0 & 0 \\ 0 & 0 & 0 & 0 \end{bmatrix}, \tag{A.8}$$

$$\boldsymbol{K}_{13} = \begin{bmatrix} 0 & 0 & 0 & 0 \\ 0 & 0 & 0 & \overline{\lambda} \\ 0 & 0 & 0 & 0 \\ 0 & \overline{\mu} & 0 & 0 \end{bmatrix}, \tag{A.9}$$

$$\boldsymbol{K}_{21} = \begin{bmatrix} 0 & 0 & 0 & 0 \\ 0 & 0 & \overline{\mu} & 0 \\ 0 & \overline{\lambda} & 0 & 0 \\ 0 & 0 & 0 & 0 \end{bmatrix}, \tag{A.10}$$

$$\boldsymbol{K}_{22} = \begin{bmatrix} 0 & 0 & 0 & 0 \\ 0 & \overline{\mu} & 0 & 0 \\ 0 & 0 & 2\overline{\mu} + \overline{\lambda} & 0 \\ 0 & 0 & 0 & \overline{\mu} \end{bmatrix}, \tag{A.11}$$

$$\boldsymbol{K}_{23} = \begin{bmatrix} 0 & 0 & 0 & 0 \\ 0 & 0 & 0 & 0 \\ 0 & 0 & 0 & \overline{\lambda} \\ 0 & 0 & \overline{\mu} & 0 \end{bmatrix}, \tag{A.12}$$

$$\boldsymbol{K}_{31} = \begin{bmatrix} 0 & 0 & 0 & 0 \\ 0 & 0 & 0 & \overline{\mu} \\ 0 & 0 & 0 & 0 \\ 0 & \overline{\lambda} & 0 & 0 \end{bmatrix}, \tag{A.13}$$

$$\boldsymbol{K}_{32} = \begin{bmatrix} 0 & 0 & 0 & 0 \\ 0 & 0 & 0 & 0 \\ 0 & 0 & 0 & \overline{\mu} \\ 0 & 0 & \overline{\lambda} & 0 \end{bmatrix}, \tag{A.14}$$

$$\boldsymbol{K}_{33} = \begin{bmatrix} 0 & 0 & 0 & 0 \\ 0 & \overline{\mu} & 0 & 0 \\ 0 & 0 & \overline{\mu} & 0 \\ 0 & 0 & 0 & 2\overline{\mu} + \overline{\lambda} \end{bmatrix}, \tag{A.15}$$
